# A novel key virulence factor, FoSSP71, inhibits plant immunity and promotes pathogenesis in *Fusarium oxysporum* f. sp. *cubense*

**DOI:** 10.1128/spectrum.02940-24

**Published:** 2025-03-25

**Authors:** Shuang Liu, Junyu Wu, Yinhui Sun, Yun Xu, Siyu Zhou, Peiping Luo, Zhibiao Wang, Daipeng Chen, Xiaofei Liang, Zhensheng Kang, Li Zheng

**Affiliations:** 1 National Key Laboratory for Tropical Crop Breeding, School of Breeding and Multiplication (Sanya Institute of Breeding and Multiplication), Hainan University, Sanya, Hainan, China; 2 Key Laboratory of Green Prevention and Control of Tropical Plant Diseases and Pests, School of Tropical Agriculture and Forestry, Ministry of Education, Hainan University, Haikou, Hainan, China; 3 School of Life and Health Sciences, Hainan University, Haikou, Hainan, China; 4 State Key Laboratory of Crop Stress Biology for Arid Areas, College of Plant Protection, Northwest A&F University, Yangling, Shaanxi, China; Southwest University, Chongqing, China

**Keywords:** *Fusarium* wilt of banana, *Fusarium oxysporum*, effector protein, pathogenicity, plant immunity

## Abstract

*Fusarium* wilt of banana (*Musa* spp.), caused by *Fusarium oxysporum* f. sp. *cubense* (*Foc*), poses a significant threat to the global banana industry. Particularly, tropical race 4 of *Foc* exhibits high pathogenicity toward the major commercial banana cultivar Cavendish, and there are no effective control measures available. Here, we characterize a novel effector protein, FoSSP71, from *Foc*, which was significantly induced during the early stages of the *Foc*4 banana interaction and could suppress BAX-triggered programmed cell death in *Nicotiana benthamiana*. Transient expression of *FoSSP71* in *N. benthamiana* leaves could weaken the upregulation expression of genes involved in the SA signaling pathway induced by flg22 and significantly reduce both reactive oxygen species bursts and callose accumulation. To verify the function of FoSSP71, a *FoSSP71* deletion mutant was created. The *FoSSP71* deletion mutant displayed a reduced growth rate in *F. oxysporum* and a marked reduction in virulence toward bananas compared to the wild type (WT). Furthermore, the expression levels of *PR3* and *PR10* were significantly downregulated in bananas infected with the *ΔFoSSP71* strain compared to bananas infected with the WT strain. These findings indicate that FoSSP71 is essential for *Foc*4 pathogenicity and plays a key virulence role during *Fusarium* invasion. Therefore, FoSSP71 presents a potential target for future *Fusarium* wilt control, offering a scientific foundation for breeding disease-resistant banana varieties and developing novel control measures.

**IMPORTANCE:**

Effector proteins are critical virulence factors for fungi, playing essential roles during the fungal infection of plants. In this study, we identified a novel effector protein, FoSSP71, which is an important regulatory protein involved in the invasion of bananas by *Fusarium oxysporum* f. sp. *cubense* race 4 (*Foc*4). Understanding its regulatory mechanisms is necessary. Our research indicates that FoSSP71 is an essential virulence factor for *Foc*4, as it suppresses plant immune responses by inhibiting the accumulation of reactive oxygen species and callose. The *Foc*4 mutant lacking *FoSSP71* showed significantly reduced pathogenicity toward bananas, demonstrating that FoSSP71 is a potential target for controlling banana wilt disease. These findings provide a scientific basis for breeding banana varieties resistant to wilt disease and for developing new disease control strategies, which are crucial for the sustainable development of the global banana industry.

## INTRODUCTION

Banana is one of the most important fruits globally, serving as both a staple food crop in many tropical and subtropical countries and a major economic commodity worldwide (1, 2). However, banana production faces threats from numerous diseases, with *Fusarium* wilt being one of the most devastating (3, 4). *Fusarium* wilt, caused by *Fusarium oxysporum* f. sp. *cubense* (*Foc*) is highly infectious. *Foc*, a member of the genus *Fusarium* under the Deuteromycetes class, is a soil-borne fungal pathogen (5–7). *Foc* exhibits significant genetic diversity and can be classified into several races based on its pathogenicity toward different banana varieties. The main physiological races include Race 1, Race 2, Race 3, and Race 4, with Tropical Race 4 posing the most severe threat to widely cultivated banana varieties, such as Cavendish (8, 9). Once Tropical Race 4 invades a banana plantation, it spreads rapidly and can devastate entire plantations. The lack of effective resistant varieties and control measures often leads to sharp declines in yield, even driving farmers to bankruptcy. The global banana industry suffers economic losses amounting to billions of dollars annually due to *Fusarium* wilt, severely impacting the socio-economic stability of affected regions (10, 11).

During pathogen–plant interactions, pathogens employ various strategies to suppress or evade the host’s immune responses. At different stages of infection, pathogens encode numerous proteins that promote fungal colonization, with effectors playing a critical role in this process. These effectors significantly impact plant defense responses, resulting in compromised plant health and growth (12–14). The genome of *F. oxysporum* is known to encode more than 1,300 secreted proteins, which alter cellular structures and metabolic pathways. Among them, the secreted in xylem (SIX) family of proteins is well-characterized, with 14 SIX proteins (SIX1–14) identified in *Fol*. Of these, SIX1 is essential for I-3-mediated resistance in tomato (*Lycopersicon esculentum*), also referred to as Avr3. The deletion of the SIX1 homolog in *Foc*4 reduces its virulence toward bananas (15, 16). The *FocSge1* gene regulates the expression of effector genes in *Foc*4 (17), while SIX8 has been shown to contribute to the virulence of *Foc*4 (18, 19). In addition to the SIX proteins that have been studied, *Foc*4 also contains many other types of effector proteins. For instance, *Foc*M35_1, a metalloprotease effector protein, enhances the virulence of *Foc4* and is upregulated during the early stages of infection (20). *Fosp9* is crucial for the full virulence of *F. oxysporum* on banana (*Musa* spp.) (21). These effector proteins are believed to play key roles in the pathogenicity of the fungus.

In our previous research, we combined bioinformatics predictions, transcriptome analysis, and transient expression in *Nicotiana benthamiana* to identify effectors in *Foc*4, revealing 80 candidate effectors that may play roles during plant infection, providing a valuable resource for studying *Foc*4 effectors (22). This study identifies FoSSP71 as a critical virulence factor in *F. oxysporum*, which enhances pathogenicity by suppressing host immune responses, including reactive oxygen species (ROS) bursts, callose deposition, and defense-related gene expression. Functional analyses reveal that FoSSP71 is essential for fungal colonization, growth, and sporulation. This study not only elucidates the critical function of FoSSP71 in the disease process but also identifies a potential molecular target for developing resistance breeding strategies and novel control measures for banana wilt disease, contributing to the sustainable development of the global banana industry.

## RESULTS

### *FoSSP71* is conserved in *Fusarium* species

**Fig 1:**
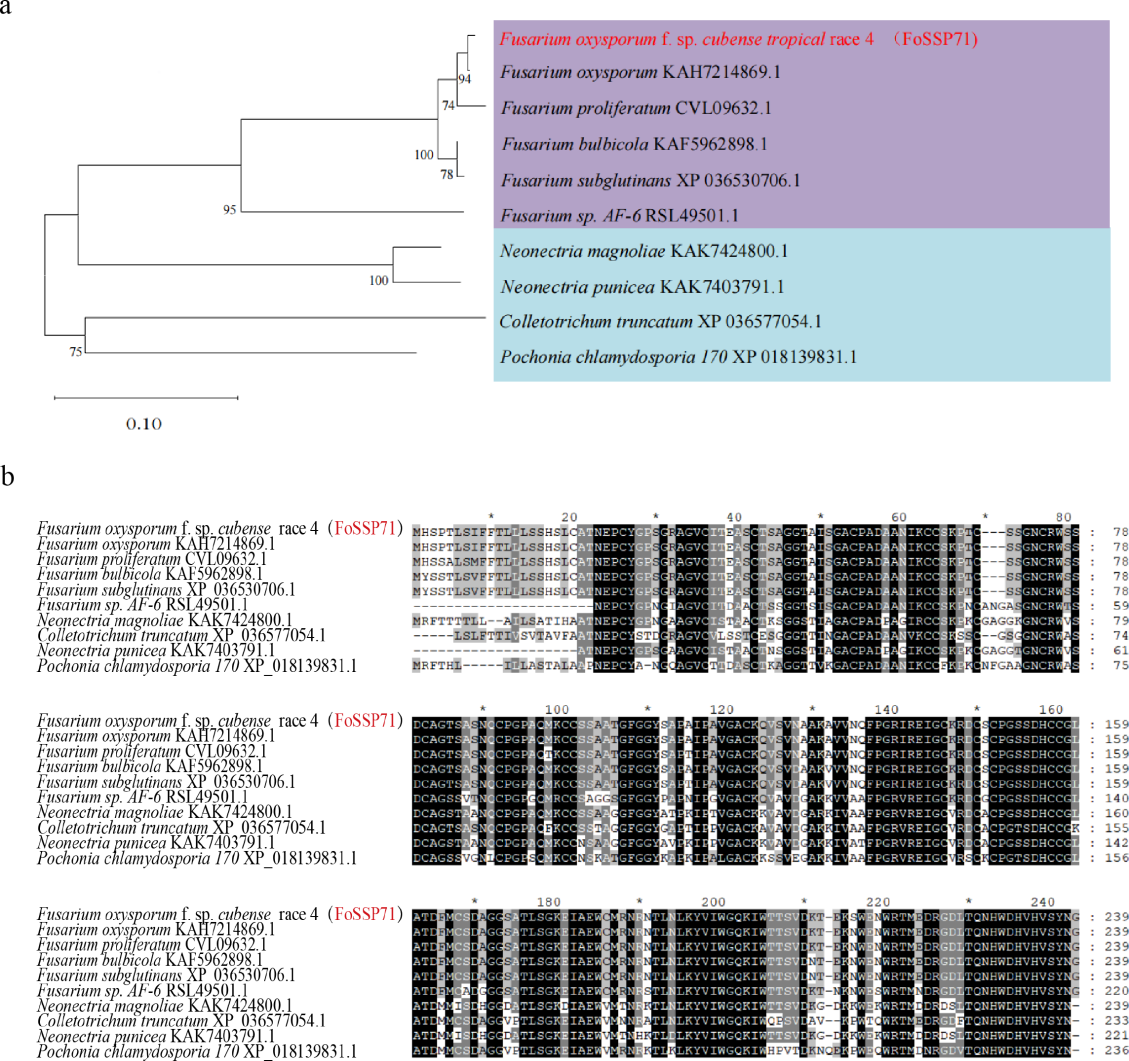
FoSSP71 phylogenetic tree and homologous protein comparison. (a) Phylogenetic analysis of the FoSSP71. The maximum likelihood tree was constructed from FoSSP71 and its immediate homologs from nine different fungal pathogens. (b) In the amino acid sequence comparison of FoSSP71 and its homologs in other species, shaded dark background indicates conserved common amino acids and dots indicate gaps in the amino acid sequence.

The *FoSSP71* gene (FOIG_04289, Gene ID: 42029464) encodes a 239-amino acid protein with an N-terminal signal peptide (SP) but lacks transmembrane domains (Fig. S1), classifying it as a classical secreted protein. The National Center for Biotechnology Information (NCBI) non-redundant protein database revealed homologs of FoSSP71 in various *Fusarium* species (Fig. 1a), including *F. oxysporum* (KAH7214869.1), *F. proliferatum* (CVL09632.1), *F. bulbicola* (KAF5962898.1), *F. subglutinans* (XP_036530706.1), and *F. sp. AF-6* (RSL49501.1).

In addition to its conservation within *Fusarium* species, homologous proteins were identified in other higher ascomycetes, such as *Colletotrichum truncatum* (XP_036577054.1), *Neonectria magnoliae* (KAK7424800.1), *N. punicea* (KAK7403791.1), and *Pochonia chlamydosporia* (XP_018139831.1). Sequence similarities ranged from 70% to 99%, with the *F. oxysporum* homolog (KAH7214869.1) sharing 99% identity with *FoSSP71*. These results indicate that *FoSSP71* is evolutionarily conserved among ascomycetes and particularly conserved within *Fusarium* species.

### FoSSP71 is a classical secreted protein that functions extracellularly in plants

**Fig 2:**
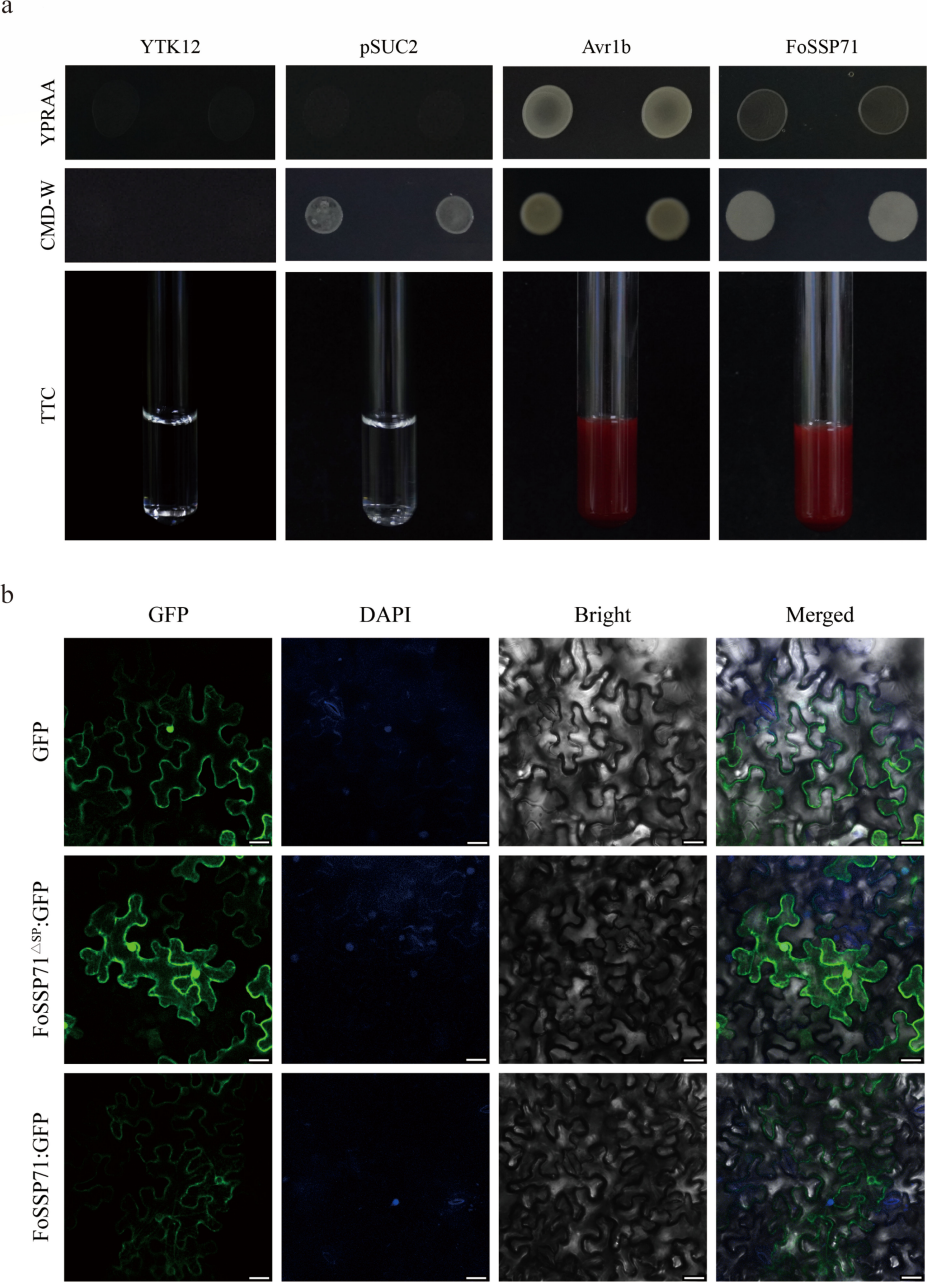
Functional validation of the FoSSP71 signal peptide and subcellular localization of FoSSP71 and FoSSP71^ΔSP^. (a) The pSUC2-*FoSSP71^sp^* vector was transformed into yeast strain YTK12, and the signaling peptide secretion function was verified on CMD-W and YPRAA media. YTK12 was a nutrient-deficient yeast strain as a blank control; pSUC2 was the YTK12 yeast strain containing the pSUC2 null load as a negative control; Avr1b was the Avr1b signaling peptide-containing pSUC2 vector of YTK12 yeast strain, as a positive control. (b) *Agrobacterium sap* containing pBin-*FoSSP71*-GFP, pBin-*FoSSP71^∆sp^*-GFP, and pBin-*GFP* was injected into leaves of *N. benthamiana* for 36 h and observed under a confocal microscope (bars  =  25 µm).

Bioinformatics analysis predicted that the N-terminus of FoSSP71 contains a 20-amino acid SP (Fig. S1); its secretory function was further validated using a yeast secretion system assay (Fig. 2a). A vector containing pSUC2-*FoSSP71^sp^* was constructed and transformed into the yeast strain YTK12. Avr1b was used as a positive control (23), while untransformed YTK12 and YTK12 transformed with pSUC2 served as negative controls. The results demonstrated that FoSSP71^sp^ enabled growth on YPRAA medium and reduced TTC solution to a red color (Fig. 2a), indicating that the N-terminal SP of FoSSP71 has secretion activity, classifying FoSSP71 as a secreted protein.

To investigate whether the SP affects the subcellular localization of FoSSP71, an *Agrobacterium*-mediated transient expression system was employed. FoSSP71-GFP and FoSSP71^ΔSP^-GFP fusion proteins were expressed in *N. benthamiana* leaves, with an empty GFP vector serving as the negative control. Laser confocal microscopy revealed distinct localization patterns: the FoSSP71-GFP fusion protein localized to the intercellular substance, while the FoSSP71^ΔSP^-GFP fusion protein was observed in both the cell membrane and the nucleus (Fig. 2b). These results suggest that the SP of FoSSP71 plays a critical role in determining its subcellular localization.

### FoSSP71 inhibits programmed cell death (PCD) in *N. benthamiana*

**Fig 3:**
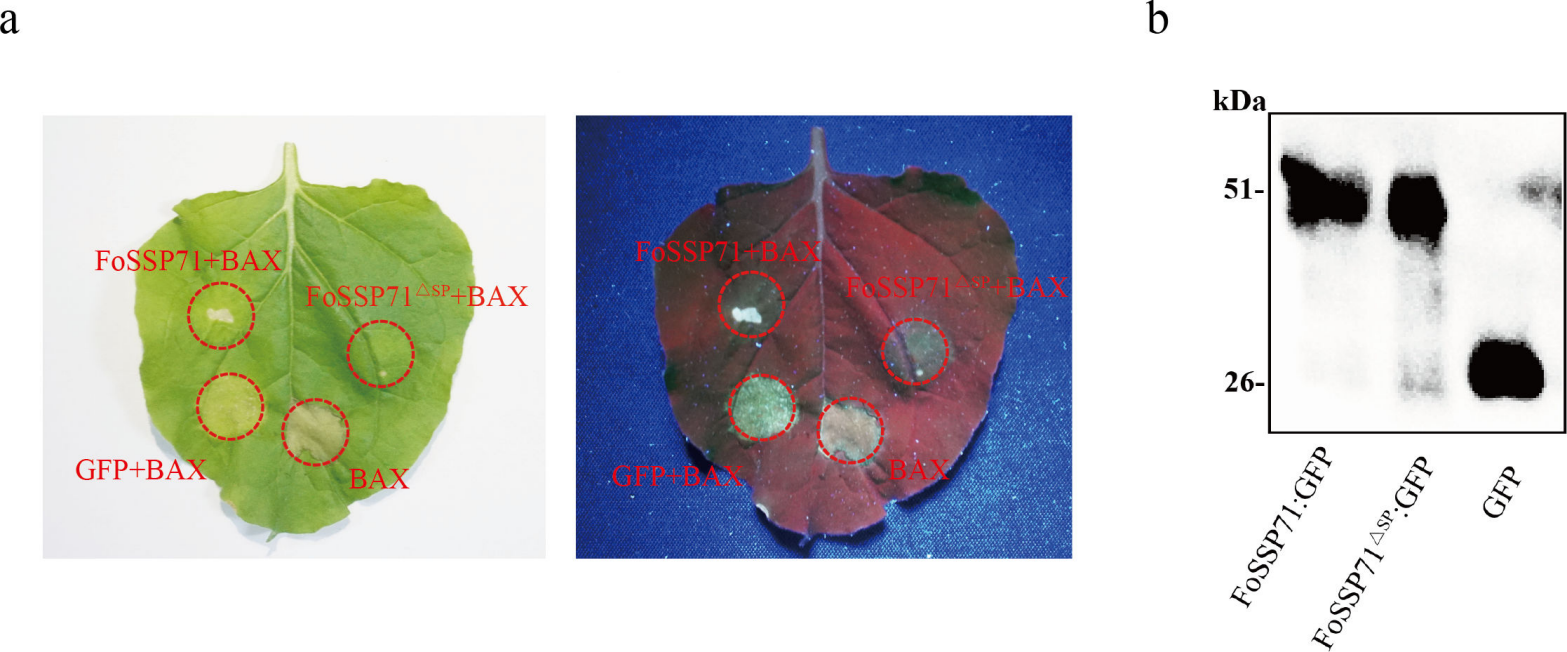
FoSSP71 and FoSSP71^ΔSP^ inhibit Bax-induced cell death in *N. benthamiana*. (a) *Agrobacterium tumefaciens* containing PVX-*FoSSP71*-GFP, PVX-*FoSSP71^ΔSP^*^-^GFP, and PVX-*eGFP* were co-injected with *A. tumefaciens* containing PVX-BAX. The black dashed areas indicate that the injection sites at 6 days after injection were irradiated with natural light and UV light, respectively. (b) Western blot analysis of FoSSP71 and FoSSP71^ΔSP^ protein expression with GFP tag. Similar results were obtained from at least three biological replicates. UV, ultraviolet.

To investigate whether FoSSP71 suppresses plant immune responses, we transiently expressed it in *N. benthamiana* and further assessed whether the presence of the SP domain affects FoSSP71’s ability to inhibit PCD. We constructed *FoSSP71* and its SP-deletion mutant, *FoSSP71^∆SP^*, into the plant transient expression vector PVX. Using an *Agrobacterium*-mediated transformation system, we injected the *Agrobacterium* cultures carrying the constructs into *N. benthamiana* leaves for transient expression. After 24 h, the PVX fusion protein was injected at the same sites. BAX, a member of the Bcl-2 family, is known to regulate apoptosis (24) and strongly induces cell death in *N. benthamiana* (25). The experimental results showed that, after 3 days of infiltration, both FoSSP71 and FoSSP71^∆SP^ effectively suppressed BAX-mediated cell death in *N. benthamiana* leaves (Fig. 3a). Western blot analysis confirmed the expression of FoSSP71 and FoSSP71^ΔSP^ fusion proteins in *N. benthamiana* (Fig. 3b).

### The FoSSP71 inhibits the expression of host defense response-related genes

**Fig 4:**
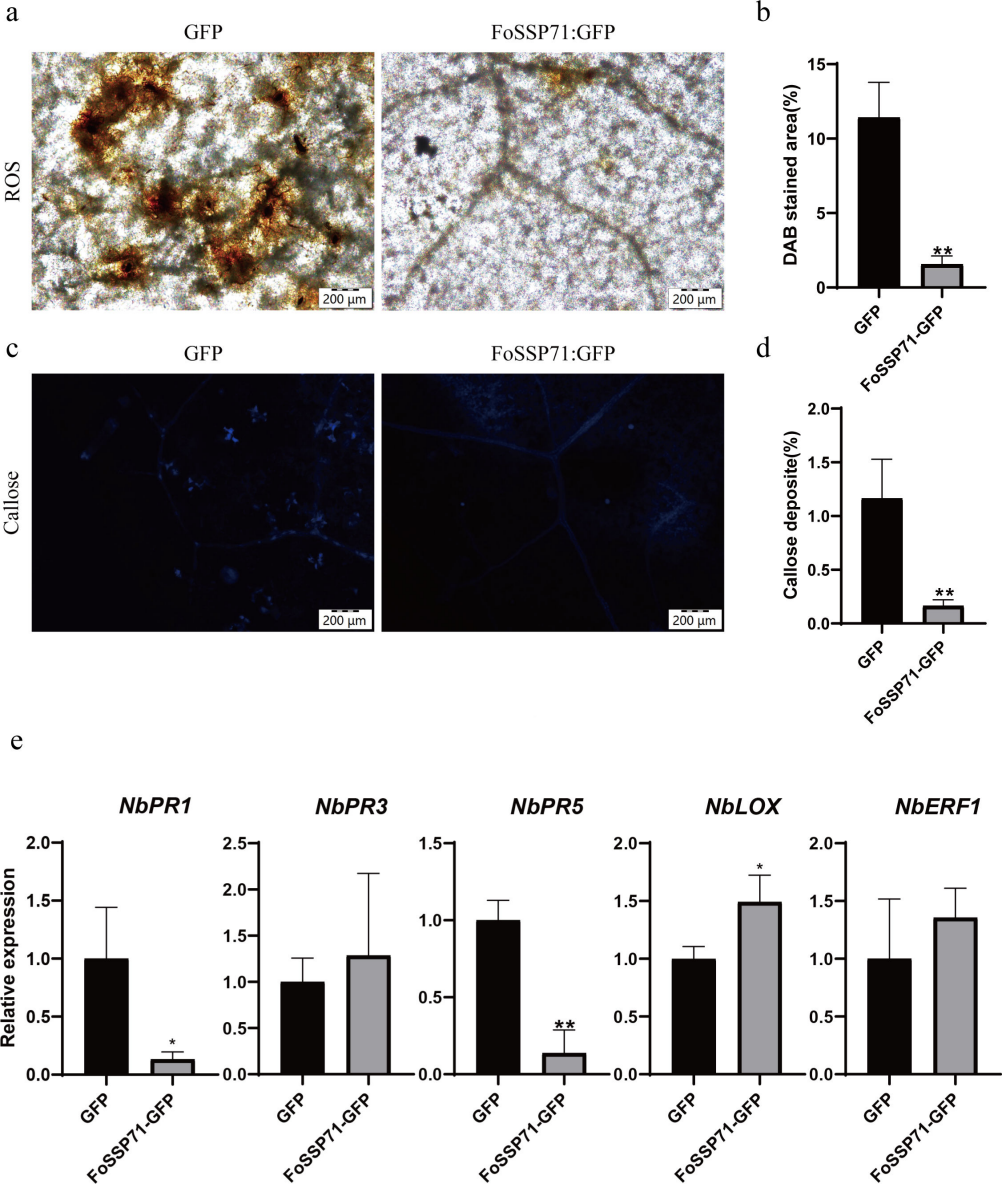
FoSSP71 inhibits flg22-triggered ROS accumulation and callosum deposition in *N. benthamiana*. (a, c) The *N. benthamiana* was infiltrated with *Agrobacterium tumefaciens*-mediated transformation and injected with flg22. After 2 days, stain with DAB and aniline blue post-infiltration (bars  = 200 µm). ROS accumulation and corpus callosum deposition induced by flg22 are inhibited by FOSSP71. (b) Percentage of H_2_O_2_ production sites. Calculate the DAB staining area using ImageJ software. (d) Percentage of callosum deposits. Calculate the aniline blue staining area using ImageJ software. (e) FoSSP71 inhibits the expression of salicylic acid pathway-related genes. Total RNA was extracted from samples collected 12 h after flg22 treatment to detect the relative transcription levels of five defense-related genes. The *NbActin* gene was used as a normalized reference gene. For (b, d, e), the data shown are means ± SD calculated from three biological replicates. Statistical analyses were performed using Student’s *t*-test. *P < 0.05; **P < 0.01. DAB, 3,3′-diaminobenzidine; ROS, reactive oxygen species; SD, standard deviation.

FoSSP71 has been observed to suppress BAX-induced PCD, leading us to hypothesize that it may inhibit key plant defense mechanisms, such as ROS bursts and callose accumulation during the early stages of plant immune responses. To test this, we transiently expressed the FoSSP71-GFP fusion protein in *N. benthamiana* leaves and treated the leaves with flg22 2 days post-expression to induce ROS production. After 12 h of flg22 treatment, ROS bursts were visualized using 3,3′-diaminobenzidine (DAB) staining, which showed a significant reduction in ROS production in FoSSP71-expressing leaves compared to controls (Fig. 4a and b). Concurrently, aniline blue staining revealed that FoSSP71 expression also markedly reduced callose deposition (Fig. 4c and d). These results suggest that FoSSP71 plays a critical role in suppressing plant immune responses by inhibiting ROS bursts and callose accumulation, thereby interfering with early defense mechanisms.

To further investigate which signaling pathways in plant immunity might be influenced by the reduced virulence of Δ*FoSSP71*, we analyzed the expression levels of several marker genes. These included *NbPR1* and *NbPR5*, which are associated with the salicylic acid (SA) signaling pathway (26); *NbPR3* and *NbLOX*, which are related to the jasmonic acid pathway; and *NbERF1*, which is involved in the ethylene pathway (27). Twenty-four hours after injecting FoSSP71 into *N. benthamiana*, we observed a significant reduction in the expression of *NbPR1* and *NbPR5* in *N. benthamiana* expressing FoSSP71 compared to those expressing only GFP. The expression of *NbLOX* showed a slight increase, while no significant changes were observed in *NbPR3* and *NbERF1* expression levels compared to control plants (Fig. 4e). These results indicate that FoSSP71 likely modulates plant immunity by downregulating the expression of genes involved in the SA signaling pathway.

### The *FoSSP71* deletion mutant leads to reduced growth rate in *F. oxysporum*

**Fig 5:**
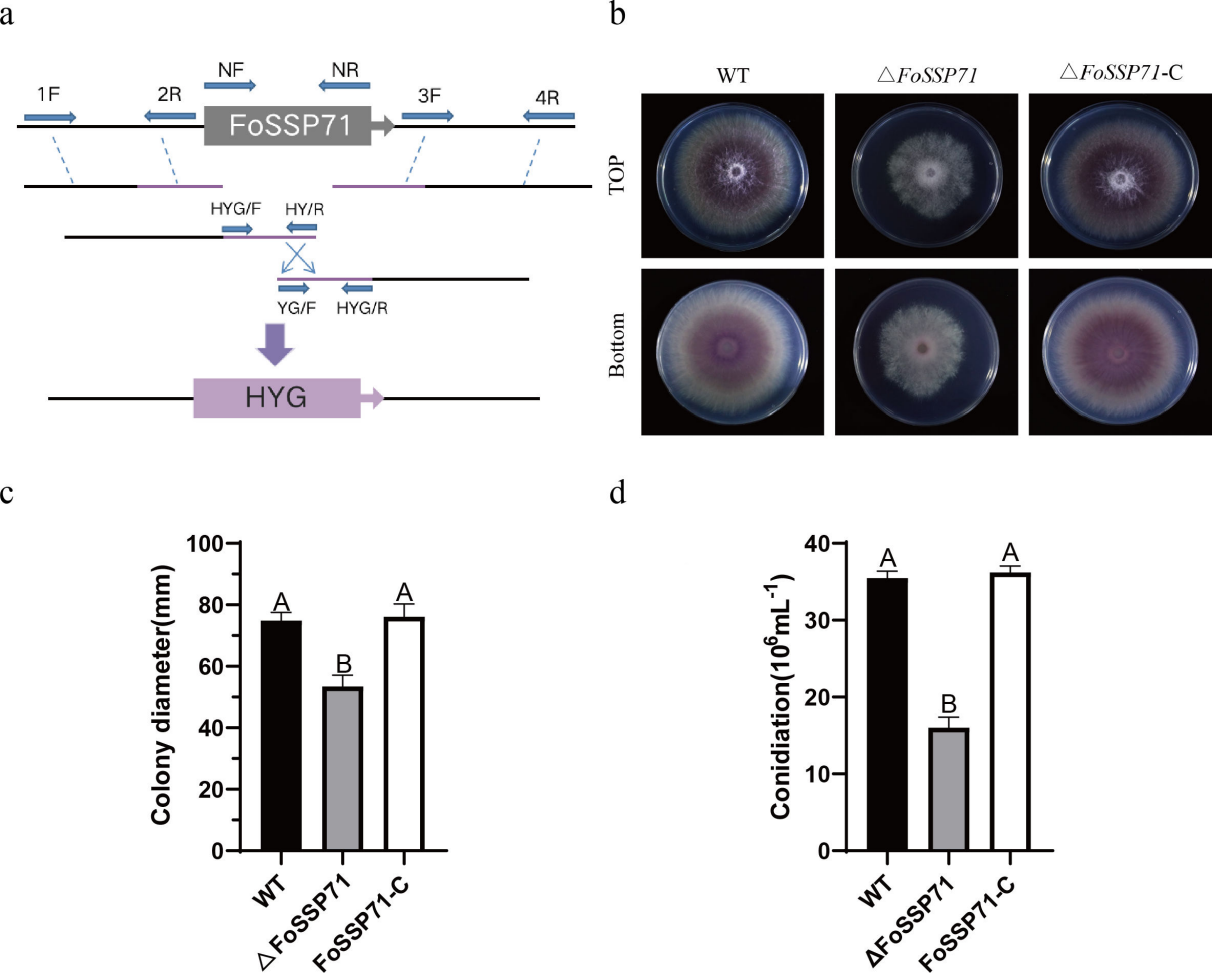
*FoSSP71* positively regulates the development of *Foc*4. (a) Replacement of the *FoSSP71* gene by homologous recombination. NF, upstream flanking region; NR, downstream flanking region. (b) The colony morphology was observed after 5 days of incubation on a PDA solid medium. (c) Colony diameter of WT, FoSSP71, and FoSSP71-C in PDB medium for 5 days. Different letters indicate significant differences (*P* <  0.05). (d) Conidia number of WT, FoSSP71, and FoSSP71-C in PDB medium for 2 days. Different letters indicate significant differences (*P* <  0.01). The data shown are means ± SD calculated from three independent experiments. Statistical analyses were performed using one-way analysis of variance and Tukey’s multiple comparison test. SD, standard deviation; WT, wild type.

Given that FoSSP71 can inhibit PCD in *N. benthamiana*, as well as suppress the expression of the SA signaling pathway, ROS burst, and callose accumulation, it serves to attenuate the plant immune response. To investigate the impact of *FoSSP71* on the pathogenicity of *Foc*, we generated a deletion mutant of *FoSSP71* using the split-marker method and protoplast transformation techniques (Fig. 5a). A *FoSSP71* mutant carrying a hygromycin resistance marker was successfully developed in *Foc*4 and further confirmed by PCR (Fig. S1). To complement the *FoSSP17* deletion mutant, we cloned the coding region of FoSSP71 into the pFL2 vector and transformed the resulting plasmid into the Δ*FoSSP71* strain through polyethylene glycol (PEG)-mediated protoplast transformation. The genetically complemented strain (*FoSSP17*-C) was validated by PCR (Fig. S1). Subsequently, we performed colony morphology identification for all three strains (Fig. 5b). The results revealed significant differences in colony morphology and growth rates between the knockout mutant Δ*FoSSP71* and the wild type (WT) as well as *FoSSP17*-C after 6 days of culturing on potato dextrose agar (PDA) medium. Compared to WT and *FoSSP17*-C, the pigmentation of Δ*FoSSP71* was notably reduced. Additionally, the colony diameter was decreased by approximately 28% compared to WT and *FoSSP17*-C (Fig. 5b and c). To confirm whether the knockout of the *FoSSP71* gene affects the conidial production of *Foc*, we analyzed the number of conidia produced by the Δ*FoSSP71* strain cultured in a PDB medium for 3 days. The conidial production in Δ*FoSSP71* decreased by approximately 45% compared to WT and *FoSSP17*-C (Fig. 5d). Therefore, our results indicate that *FoSSP71* is essential for fungal growth and development.

### The *FoSSP71* deletion mutant reduced the virulence of *Foc* toward bananas

**Fig 6:**
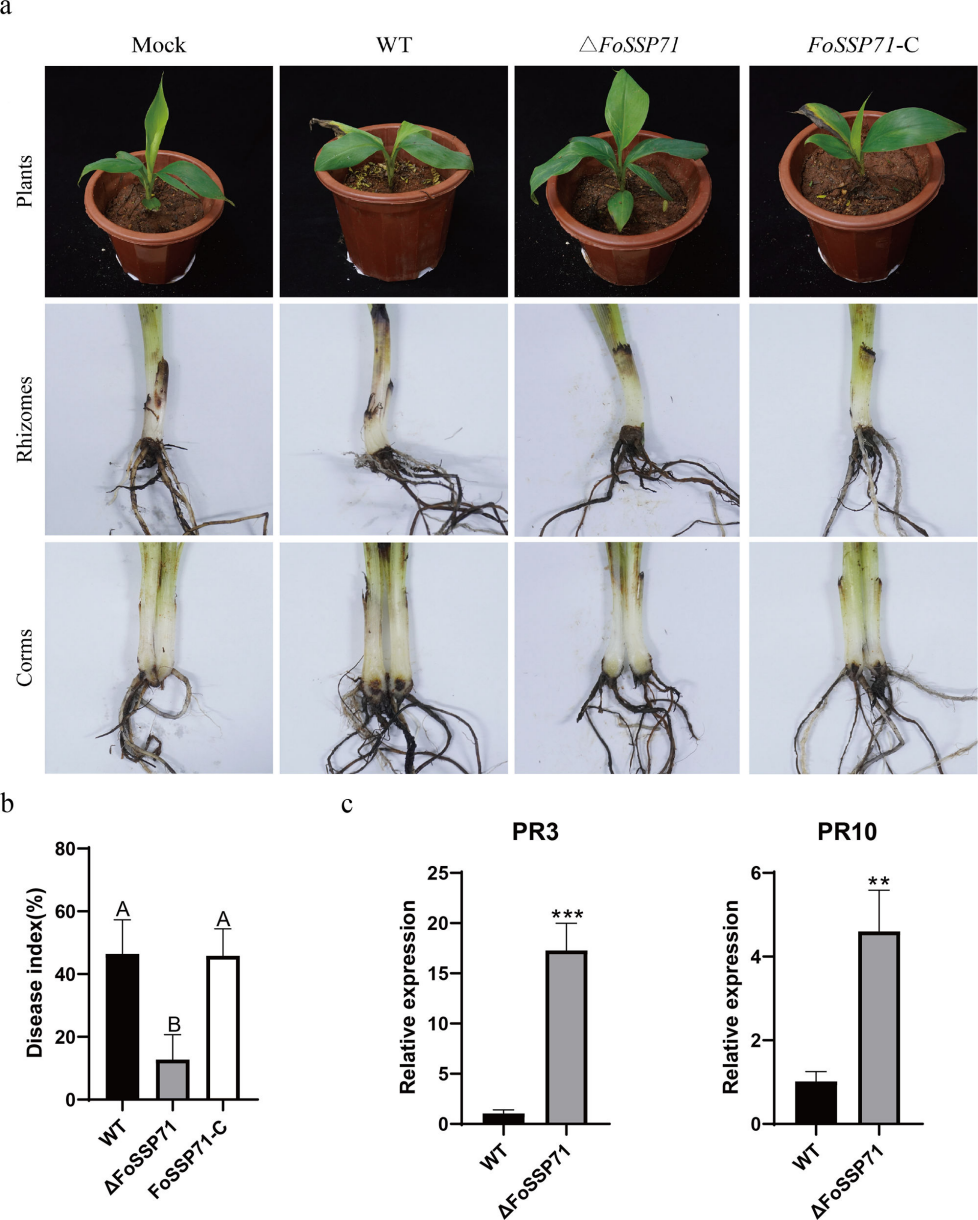
Absence of *FoSSP71* reduces *Foc*4 toxicity to banana roots and root stocks. (a) Disease phenotype of banana after 30 days of inoculation with WT, *FoSSP71,* and *FoSSP71*-C, respectively. Banana plants at the full four-leaf stage were inoculated with *FoSSP71* deletion mutants and complementary strains. Photographs of the young plants were taken 30 days after inoculation. The experiment was repeated three times with 15 replicates for each of the three strains. (b) Disease index of banana inoculation with level 3 and level 2 and level 3, respectively. (c) qRT-PCR assay for defense genes (*PR*). The total RNA of banana roots inoculated with WT and ΔFOSSP71 strains was extracted for 3 days, and the internal reference gene was the *Action* gene of banana, and the expression level of the *PR* gene in bananas inoculated with the WT strain was set to 1. For (b, c), the data shown are means ± SD calculated from three biological replicates. Statistical analyses were performed with one-way analysis of variance, Tukey’s multiple comparison test, and Student’s *t*-test. Different letters indicate significant differences (*P* < 0.05). *P < 0.05; **P < 0.01. PR, pathogenesis-related; SD, standard deviation; WT, wild type.

To assess the role of *FoSSP71* in the virulence of *Foc*4, conidial suspensions of WT, Δ*FoSSP71*, and *FoSSP71*-C strains were used to inoculate the banana roots. After 30 days, plants infected with the FoSSP71 deletion mutant exhibited significantly attenuated disease symptoms, including fewer yellowed leaves and reduced black-brown discoloration of pseudo-stems, compared to those inoculated with WT and *FoSSP71*-C. The disease index was notably lower in plants inoculated with Δ*FoSSP71* (Fig. 6a and b; Table S2). Additionally, quantitative real-time polymerase chain reactionq (RT-PCR) analysis revealed a marked upregulation of *pathogenesis-related* (*PR*) gene expression in bananas infected with the Δ*FoSSP71* strain relative to those infected with the WT strain (Fig. 6c). These findings provide compelling evidence that *FoSSP71* is a critical virulence factor in *Foc*4, facilitating disease progression and suppressing host defense responses.

## DISCUSSION

The infection of bananas by *Foc*4 is a complex process involving a series of intricate interactions between the fungus and its host (28). During this process, the fungus and the plant engage in a continuous battle and co-evolution. The plant immune system typically comprises two layers of defense mechanisms. The first layer involves the recognition of pathogen-associated molecular patterns (PAMPs). For example, plant transmembrane pattern recognition receptors (PRRs) can recognize chitin PAMPs in flagellin proteins, thereby triggering PAMP-triggered immunity (PTI) (29, 30). However, pathogens do not stop their infection process at this point; they secrete effector proteins to interfere with the PTI process, leading to enhanced susceptibility of the host plant (effector-triggered susceptibility) (31). This, in turn, activates the plant’s second layer of defense, known as effector-triggered immunity (32, 33). This process is often accompanied by a localized hypersensitive response (HR) and ROS burst. To date, only a few effector proteins essential for the virulence of *Foc*4 have been identified.

In this study, we identified a new effector protein, FoSSP71, and investigated its potential role in inducing or inhibiting cell death in the non-host plant *N. benthamiana*. The results showed that FoSSP71 could inhibit BAX-mediated cell death, ROS production, and callose accumulation. Furthermore, we observed that transient expression of FoSSP71 significantly reduced the expression levels of two defense-related genes (*NbPR1* and *NbPR3*) in *N. benthamiana* leaves. In the absence of *FoSSP71*, the pathogen’s growth, development, and virulence were adversely affected, indicating that FoSSP71 is crucial for the fungal pathogen.

Effector proteins can suppress cell death responses triggered by R proteins (34). For example, effector proteins secreted by *Foc* inhibit PCD in plant cells, preventing HR and allowing the pathogen to continue growing and spreading within the plant (35). Our results suggest that FoSSP71 may promote fungal invasion by suppressing the plant’s defense responses. Additionally, we found that FoSSP71 can inhibit BAX-induced cell death regardless of whether it contains a SP, indicating that the SP is not necessary for its ability to suppress cell death in *N. benthamiana*. However, subcellular localization experiments revealed that FoSSP71, with or without the SP, exhibits different intracellular localization patterns. In summary, these findings suggest that FoSSP71 functions as a virulence factor of *Foc*4 and may relocate within the host cell to exert its function.

### Conclusion

In summary, this study employed molecular biology, bioinformatics, and functional validation experiments to elucidate the role of FoSSP71 as a key virulence factor in *Foc*4. Using homologous recombination technology, we generated *FoSSP71* knockout and complementation mutants and analyzed their colony morphology, growth rate, pathogenicity, and gene expression levels. The main findings are as follows: (i) FoSSP71 exhibits significant evolutionary conservation. (ii) Its SP determines subcellular localization and suppresses plant immune responses, including ROS bursts, callose deposition, and the expression of defense-related genes. (iii) Knockout of *FoSSP71* significantly reduced the pathogenicity of Foc4 toward bananas and negatively impacted fungal development, resulting in a 28% reduction in colony diameter and a 45% decrease in conidial production. This study demonstrates that FoSSP71 is an indispensable virulence factor during *Fusarium* infection, providing a potential target for developing resistant banana varieties and effective disease control strategies. These findings offer a scientific basis for the development of FoSSP71-targeted inhibitors, the breeding of resistant banana cultivars, and the establishment of integrated disease management systems, with significant agricultural application potential.

## MATERIALS AND METHODS

### Bioinformatics analysis

The DNA sequence of *FoSSP71* (FOIG_04289) was downloaded from NCBI (genome sequence: XP_031067898.1). *FoSSP71* homolog sequences were identified through the NCBI NR and Ensemble Fungi databases. The phylogenetic tree was constructed using MEGA11 with the maximum likelihood method (36). Amino acid alignments were performed using Clustal W, and the alignment results were edited with GeneDoc. The SignalP 6.0 Server (http://www.cbs.dtu.dk/services/SignalP/) was used to identify SPs in the protein (37). Transmembrane domain analysis of the protein was predicted using the online software TMHMM Server v.2.0 (http://www.cbs.dtu.dk/services/TMHMM/) (38). Subcellular localization analysis of the protein was performed using the online analysis tool Deep-Loc1.0 (DeepLoc 1.0—DTU Health Tech—Bioinformatic Services).

### Strains and plant growth conditions

The *Foc*4 strain was grown in the dark on PDA medium at 28°C. The *FoSSP71* gene knockout mutant was generated in *Foc*4 using the split-marker method (39); the upstream and downstream sequences of *FoSSP71* were fused with partial fragments of HYG through overlapping PCR. The knockout mutants and complemented strains were cultivated on a PDA medium containing 60 µg/mL hygromycin B or G418, respectively (40). *N. benthamiana* was grown in a growth chamber at 24°C with a 16 h light/8 h dark cycle. “Cavendish” bananas (AAA, Brazil) with five to six leaves were planted in a greenhouse at 28 ± 2°C with a 16 h light/18 h dark cycle. *Escherichia coli* (DH5α) and *Agrobacterium tumefaciens* (GV3101) were stored at −80°C and grown in Luria-Bertani (LB) broth at 37°C and 28°C, respectively. The yeast strain YTK12 was grown in yeast extract peptone dextrose medium at 30°C.

### RNA extraction and qRT-PCR analysis

Total RNA was extracted from infected banana roots or treated *N. benthamiana* leaves using the Eastep Super Total RNA Extraction Kit (Promega Biotech) according to the manufacturer’s instructions. First-strand complementary DNA (cDNA) was synthesized using the Revert Aid First-Strand cDNA Synthesis Kit (Thermo Fisher Scientific) following the manufacturer’s protocol. Quantitative RT-PCR was performed using the QuantStudio 5 Real-Time PCR System with SuperReal PreMix Plus (TianGen Biotech) according to the manufacturer’s guidelines. *NbActin* (AFD62804.1) was used as a reference gene for normalization. Gene expression levels were analyzed using the QuantStudio Design & Analysis Software v1.5.2 by the comparative threshold cycle (Ct) method. At least three biological replicates were performed.

### Plasmid construction

To investigate whether FoSSP71 can induce an HR in *N. benthamiana*, the coding region of *FoSSP71* and the coding region without the SP (*FoSSP71^ΔSP^*) were amplified from cDNA of *Foc*4 using Phusion Plus DNA Polymerase (Thermo Fisher Scientific) and cloned into the plant expression vector pBin-*eGFP*. The same sequences were PCR amplified and cloned into the pSUC2 vector using restriction sites and the ClonExpress II One Step Cloning Kit (Vazyme Biotech) to verify the secretion activity of the SP. The coding region and promoter sequence of *FoSSP71* were amplified from WT genomic DNA and cloned into the pFL2 vector to obtain the complementing vector pFL2-*FoSSP71*.

### SP secretion activity validation

Recombinant vectors pSUC2-*FoSSP71* and pSUC2-*FoSSP71^ΔSP^* were constructed and transferred into the yeast strain YTK12. Positive colonies were screened from all transformants on CMD-W medium (0.08% tryptophan dropout supplement, 0.65% amino acid-free yeast nitrogen base, 2% sucrose, 0.1% glucose, and 2% agar). Successful transformants were cultured on a YPRAA medium to verify their secretion activity. To further test the secretion activity of the SP, the reduction of 2,3,5-triphenyl tetrazolium chloride to insoluble red 1,3,5-triphenylformazan was monitored (41).

### Cultivation conditions and fungal transformation

Gene replacement was performed based on homologous recombination. Using WT DNA of *Foc* and the PEX-2 plasmid as templates, specific primers *FoSSP71*/1F and *FoSSP71*/2R (upstream); *FoSSP71*/3F and *FoSSP71*/4R (downstream); and HYG/F and HY/R; YG/F and HYG/R were used with Phanta High-Fidelity DNA Polymerase to amplify the target bands (Table S1). As described, fusion fragments for transformation were generated by overlapping PCR. The purified fragments for protoplast transformation were obtained using a gel extraction kit (Omega) according to the manufacturer’s instructions. Mycelia were digested in an enzyme solution (25 g/L Driselase and 5 g/L lysing enzyme in 1.2 M KCl) until the protoplast concentration reached 10^7^ protoplasts/mL. DNA fragments or plasmids intended for transformation were mixed with the protoplasts and incubated on ice for 20 min. Then, 1 mL of PTC solution (0.6 M KCl, 50 mM Tris-HCl pH 8.0, 50 mM CaCl_2_, and 40% PEG 4000) was added to the mixture, and incubation continued at room temperature for 30 min. The mixture was then transferred to a TB3 medium for further incubation for 20 h. The transformed mixture was inoculated onto a 15 mL PDA medium containing 50 µg/mL hygromycin B or G418, followed by overlaying with PDA agar containing 75 µg/mL hygromycin B or G418. Transformants with resistance to hygromycin B or G418 were identified by PCR.

### Transient expression assay in *N. benthamiana*

Recombinant vectors pBin-*FoSSP71* and pBin-*FoSSP71^ΔSP^* were transferred into *A. tumefaciens* GV3101 and cultured in LB medium containing 20 mg/mL rifampicin and 50 mg/mL kanamycin for 48 h. Bacteria were harvested and resuspended in injection buffer (1% MgCl_2_·6H_2_O, 1% MES, and 150 µM acetosyringone) to an optical density (OD_600_) of 1.0. The suspension was incubated at room temperature for 2–3 h to induce plasmid release. *N. benthamiana* plants at the four-leaf stage were selected, and the injection buffer was infiltrated into the leaf abaxial surface. An empty pBin-*eGFP* vector was used as a negative control, and a vector containing BAX was used as a positive control. Six days after inoculation, leaf cell death symptoms were observed and photographed under ultraviolet light (wavelength 365 nm).

### Protein extraction and western blot

Total protein from *N. benthamiana* was extracted using Plant Total Protein Extraction Buffer (Sangon Biotech) according to the manufacturer’s instructions. Denatured proteins were separated by 12.5% sodium dodecyl sulfate-polyacrylamide gel electrophoresis (SDS-PAGE ) and then transferred to a 0.22 µm polyvinylidene fluoride (PVDF) membrane. Proteins on the PVDF membrane were detected using rabbit anti-GFP pAb and Horseradish Peroxidase-conjugated (HRP-conjugated) goat anti-rabbit IgG (Abclonal Biotech).

### Phenotypic characteristics of *Foc* mutants

*ΔFoSSP71*, WT, and *FoSSP71*-C strains were inoculated on PDA plates and incubated at 28°C for 7 days. The growth rate of the fungal mycelium was tracked by measuring its expansion daily. WT, *FoSSP71*, and *FoSSP71*-C strains were also cultured in PDB medium at 28°C. After 4 days, cultures containing at least 10^7^ conidia/mL were collected (42). The roots of banana seedlings were cleaned, wounded with a blade, and then immersed in the prepared conidial suspension for 2 h. The seedlings were then transplanted into pots, with 100 mL of conidial suspension added to each pot.

To assess the severity of disease, the corm of infected banana seedlings were sliced open 30 days post-infection to measure the lesion area. The lesion area is a key indicator for determining disease severity, with each plant classified into one of the five levels from 0 to 4 (0 representing no lesions on the corm; 1 representing lesions covering 1–10% of the corm; 2 representing lesions covering 11–30% of the corm; 3 representing lesions covering 31–50% of the corm; and 4 representing lesions covering more than 50% of the corm). The disease index of infected banana seedlings was calculated using the following formula: Disease Index (%) = [Σ (Severity Level × Number of Plants at that Level)/(4 × Total Number of Plants Evaluated)] × 100% (43).

### Subcellular localization in *N. benthamiana* leaves

Recombinant vectors pBin-*FoSSP71* and pBin-*FoSSP71^ΔSP^* were resuspended in a buffer solution and adjusted to an OD_600_ of 0.01. The *Agrobacterium* solution was then injected into *N. benthamiana* leaf tissue using a syringe. After 48 h of treatment, leaf samples were photographed and observed using a laser confocal microscope, with DAPI staining performed to label the cell nuclei. Samples treated with the empty vector served as the negative control.

### ROS staining and callose deposition detection

Both flg22 and chitin are known to activate pattern-triggered immunity (PTI) in plants (44, 45). Flg22, as a microbe-associated molecular pattern, can be recognized by PRRs on the surface of plant cells, thereby triggering PTI, which leads to ROS bursts and callose accumulation (46, 47). First, flg22 was co-injected with *Agrobacterium* solution into *N. benthamiana* leaves for 3 days. In parallel, aniline blue staining was performed to assess callose deposition (48). The leaves are stained with DAB in the dark for 12 h. The DAB staining highlights ROS by producing a brown color where ROS are present. Following staining, the leaves are decolorized in 95% ethanol until they become semi-transparent. This step helps to enhance the visibility of the brown staining, making it easier to capture and analyze the results (49).

To detect callose deposition, the leaves are first boiled in 95% ethanol to remove pigments and clear the tissue. This decolorization step is crucial to ensure that callose deposition is clearly visible against the leaf tissue. After decolorization, the leaves are stained in 1% aniline blue solution, protected from light, for at least 2 h. Aniline blue specifically binds to callose, which is a polysaccharide involved in plant defense responses. The leaves are then examined under a microscope to observe and record the callose deposition, which appears as bright blue spots indicating areas of callose accumulation (50).

### Statistical analysis

All experiments, the data are presented as the mean ± standard deviation calculated from three biological replicates. Statistical analyses were performed using one-way analysis of variance (ANOVA), Tukey’s multiple comparison test, and Student’s *t*-test. GraphPad Prism 5.0 software was used for data visualization and comparisons among multiple groups using one-way ANOVA. A *P* value < 0.05 was considered statistically significant, with *P < 0.05, **P < 0.01, and ***P < 0.001.

## SUPPLEMENTAL MATERIAL

Figure S1 spectrum.02940-24-s0001.pdf Bioinformatics analysis.

Figure S2 spectrum.02940-24-s0002.pdf PCR detection of FoSSP71 gene knockout.

Tables S1 spectrum.02940-24-s0003.pdf Polynucleotide chain reaction primers used in this study.

Tables S2 spectrum.02940-24-s0004.pdf Severity of infection of banana seedlings by different hyphal mutants.

ASM does not own the copyrights to Supplemental Material that may be linked to, or accessed through, an article. The authors have granted ASM a non-exclusive, world-wide license to publish the Supplemental Material files. Please contact the corresponding author directly for reuse.

## Note

Shuang Liu andJunyu Wu contributed equally to this work. Author order was determined both alphabetically and in order of increasing seniority.
