# Supplementary material for: A novel key virulence factor, FoSSP71, inhibits plant immunity and promotes pathogenesis in Fusarium oxysporum f. sp. cubense
Source: Microbiol Spectr. 2025 Mar 25;13(5):e02940-24. doi: 10.1128/spectrum.02940-24 (PMC12054145; doi:10.1128/spectrum.02940-24)
Supplement: Figure S1 — Bioinformatics analysis. [file spectrum.02940-24-s0001.pdf]

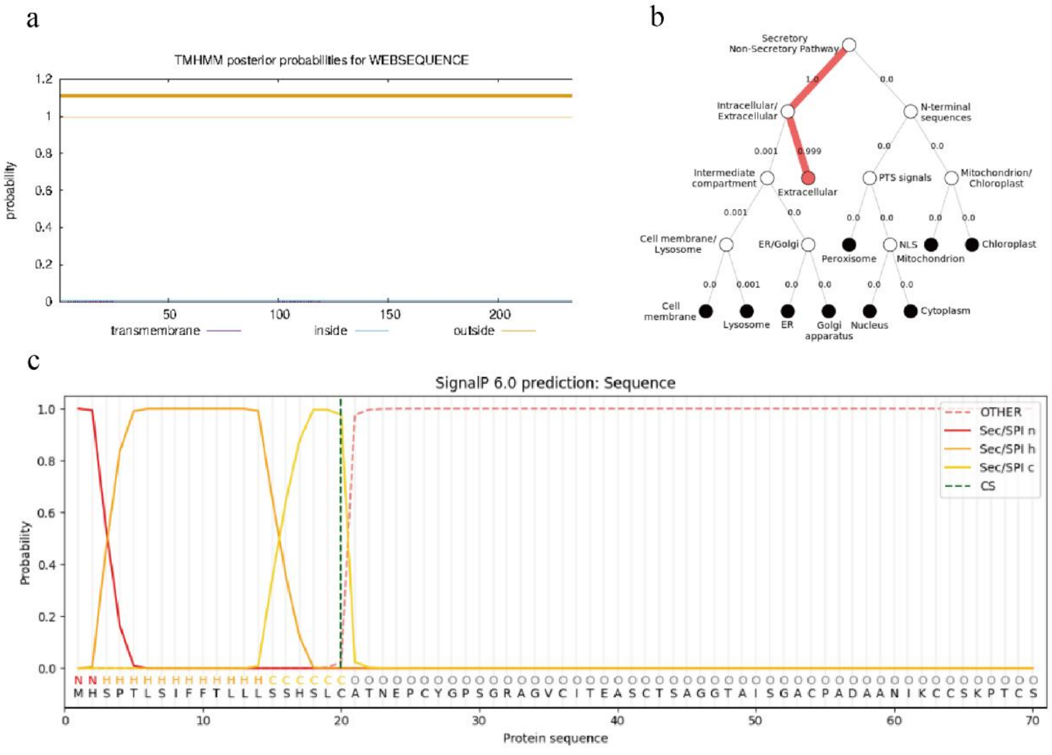


**FIG S1** Bioinformatics analysis (a) Protein transmembrane domain analysis, Forecast with the online software TMHMM Server v.2.0. (b) Subcellular localization analysis of proteins, Utilize the online analysis software Deep-Loc 1.0 (c) Signal peptide analysis, Utilize the online software SignalP 6.0 Server.
