# Supplementary material for: A novel key virulence factor, FoSSP71, inhibits plant immunity and promotes pathogenesis in Fusarium oxysporum f. sp. cubense
Source: Microbiol Spectr. 2025 Mar 25;13(5):e02940-24. doi: 10.1128/spectrum.02940-24 (PMC12054145; doi:10.1128/spectrum.02940-24)
Supplement: Figure S2 — PCR detection of FoSSP71 gene knockout. [file spectrum.02940-24-s0002.pdf]

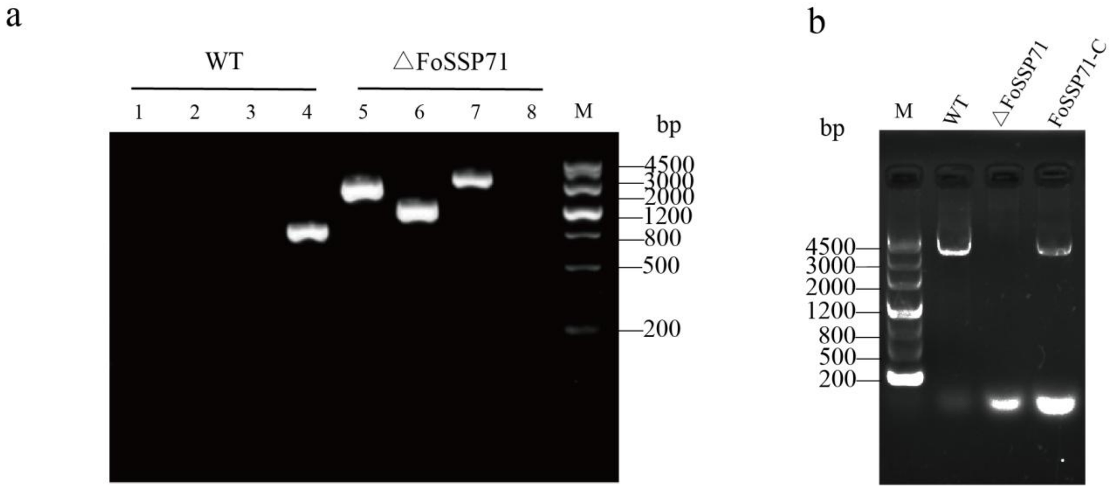

FIG S2 PCR detection of *FoSSP71* gene knockout. (a) Based on the upstream and downstream sequences of *FoSSP71*, the primers *FoSSP71*/1F and 2R, *FoSSP71*/3F and 4R were designed, and the upstream and downstream fragments were amplified with wild-type strain WT as the template, with the upstream fragment size of 1382 bp and the downstream fragment size of 1488 bp. Based on hygromycin, primers HYG/F, HY/R, YG/F and HYG/r were designed, and the HY and YG fragments were amplified by the vector PEX-2 template, with the HY fragment being 767 bp and the YG fragment being 924 bp.After the fragment was knocked out, the knockout mutants were verified with hygromycin primers, FoSSP71 internal primers were verified as band-free, WT was verified with hygromycin primers, and FoSSP71 internal primers were verified as band-free（Table S1）.（b）*FoSSP71-C* was obtained by *FoSSP71*-HB-F and *FoSSP71*-HB-R. Lane M, DL4500 marker.
