# Supplementary material for: A novel key virulence factor, FoSSP71, inhibits plant immunity and promotes pathogenesis in Fusarium oxysporum f. sp. cubense
Source: Microbiol Spectr. 2025 Mar 25;13(5):e02940-24. doi: 10.1128/spectrum.02940-24 (PMC12054145; doi:10.1128/spectrum.02940-24)
Supplement: Tables S1 — Polynucleotide chain reaction primers used in this study. [file spectrum.02940-24-s0003.pdf]

Table S1. Polynucleotide chain reaction primers used in this study.

**Primer names**	**Sequence（5’-3’）**
FoSSP71/1F	TTCGCCTATCCCGCTTTCTAC
FoSSP71/2R	TTGACCTCCACTAGCTCCAGCCAAGCC<br>TCTGCCGGTGATCTTCTTCAGC
FoSSP71/3F	CAAAGGAATAGAGTAGATGCCGACC<br>GCTGCCTCAACTGTCTCTCCA
FoSSP71/4R	TCATGGGTTGCGGATGGCTTC
FoSSP71N/F	CGCTAGGACACTGGAGGTATGT
FoSSP71N/R	GCTTGTTTGCCCTCACTGC
FoSSP71U/F	AATAGGGCCAACAACTCCGAC
FoSSP71D/R	GAAGCCTCGATTGTCCGATCCA
HYG/F	GGCTTGGCTGGAGCTAGTGGAGGTCAA
HYG/R	CGGTCGGCATCTACTCTATTCCTTTG
HY/F	GATGTAGGAGGGCGTGGATATGTCCT
YG/R	GTATTGACCGATTCCTTGCGGTCCGAA
FoSSP71-HB-F	GAATTGGGTACTCAAATTGGCGTGATTGTCGCAACCGGAA
FoSSP71-HB-R	CAGCTCCTCGCCCTTGCTCACGGAAGCCAGAGGCTCCAGTACA
pBin-FoSSP71-F	ATTTACGAACGATAGGGTACCATGCACTCCCCAACT
pBin-FoSSP71-R	TCCGTCGACCCCGGGGGTACCACCATTGTAAGAGAC
pBin-FoSSP71$^{\triangle SP}$-F	ATTTACGAACGATAGGGTACCATGGCTACCAACGAGCCC
pBin-FoSSP71$^{\triangle SP}$-R	TCCGTCGACCCCGGGGGTACCACCATTGTAAGAGAC
pSUC2-FOSSP71-F	CGGAATTTTAATTAAGAATTCATGCACTCCCCAACTCTATCCA
pSUC2-FOSSP71-R	CACTATAGGGAGAACCTCGAGGCAGAGGGAATGGGAGGATAG
pGR107-FOSSP71-F	AGGTCAGCACCAGCTAGCATCGATATGCACTCCCCAACT
pGR107-FOSSP71-R	GACGTCTTTAAATTTATCGATACCATTGTAAGAGAC
pGR107-FoSSP71$^{\triangle SP}$-F	AGGTCAGCACCAGCTAGCATCGATATGGCTACCAACGAGCCC
pGR107-FoSSP71$^{\triangle SP}$-R	GACGTCTTTAAATTTATCGATACCATTGTAAGAGAC
qRT-NbPR1-F	GATGCCCATAACACAGCTCGTGC
qRT-NbPR1-R	GCCTCTATAATTACCTGGAGGATC
qRT-NbPR3-F	CCAGAGTGACAGATATTA
qRT-NbPR3-R	GCCCTGGCCGAAGTTCCT
qRT-NbPR5-F	GTCAACCAATGCACCTAC
qRT-NbPR5-R	GGTGGATCATCCTGTGGA
qRT-NbLOX-F	CTCGTAGAGGCAGGACA

qRT-NbLOX-R	TCGTCCCTTGGCACATA
qRT-NbERF1-F	GCGGTTCAAAGGCTCA
qRT-NbERF1-R	ACAGCCTTTCTTCTCCGTT
NbActin-F	TGTGAAGGAGAAGTTGGCT
NbActin-R	GAATCTCTCAGCACCAATG
