# Supplementary material for: A novel key virulence factor, FoSSP71, inhibits plant immunity and promotes pathogenesis in Fusarium oxysporum f. sp. cubense
Source: Microbiol Spectr. 2025 Mar 25;13(5):e02940-24. doi: 10.1128/spectrum.02940-24 (PMC12054145; doi:10.1128/spectrum.02940-24)
Supplement: Tables S2 — Severity of infection of banana seedlings by different hyphal mutants. [file spectrum.02940-24-s0004.pdf]

Table S2 Severity of infection of banana seedlings by different hyphal mutants

Grade	Wild Type (wt)	ΔFoSSP71 (Knockout)	FOSSP71-C (Complemented)
Grade I	0	8	0
Grade II	9	5	4
Grade III	7	4	8
Grade IV	10	3	10
